# Co-Inoculation with Rhizobia and AMF Inhibited Soybean Red Crown Rot: From Field Study to Plant Defense-Related Gene Expression Analysis

**DOI:** 10.1371/journal.pone.0033977

**Published:** 2012-03-19

**Authors:** Xiang Gao, Xing Lu, Man Wu, Haiyan Zhang, Ruqian Pan, Jiang Tian, Shuxian Li, Hong Liao

**Affiliations:** 1 State Key Laboratory for Conservation and Utilization of Subtropical Agro-Bioresources, Root Biology Center, South China Agricultural University, Guangzhou, China; 2 Laboratory of Bacteria and Fungicides, South China Agricultural University, Guangzhou, China; 3 Crop Genetics Research Unit, United States Department of Agriculture - Agricultural Research Service, Stoneville, Mississippi, United States of America; University of Wisconsin-Milwaukee, United States of America

## Abstract

**Background:**

Soybean red crown rot is a major soil-borne disease all over the world, which severely affects soybean production. Efficient and sustainable methods are strongly desired to control the soil-borne diseases.

**Principal Findings:**

We firstly investigated the disease incidence and index of soybean red crown rot under different phosphorus (P) additions in field and found that the natural inoculation of rhizobia and arbuscular mycorrhizal fungi (AMF) could affect soybean red crown rot, particularly without P addition. Further studies in sand culture experiments showed that inoculation with rhizobia or AMF significantly decreased severity and incidence of soybean red crown rot, especially for co-inoculation with rhizobia and AMF at low P. The root colony forming unit (CFU) decreased over 50% when inoculated by rhizobia and/or AMF at low P. However, P addition only enhanced CFU when inoculated with AMF. Furthermore, root exudates of soybean inoculated with rhizobia and/or AMF significantly inhibited pathogen growth and reproduction. Quantitative RT-PCR results indicated that the transcripts of the most tested pathogen defense-related (PR) genes in roots were significantly increased by rhizobium and/or AMF inoculation. Among them, *PR2*, *PR3*, *PR4* and *PR10* reached the highest level with co-inoculation of rhizobium and AMF.

**Conclusions:**

Our results indicated that inoculation with rhizobia and AMF could directly inhibit pathogen growth and reproduction, and activate the plant overall defense system through increasing PR gene expressions. Combined with optimal P fertilization, inoculation with rhizobia and AMF could be considered as an efficient method to control soybean red crown rot in acid soils.

## Introduction

Soybean (*Glycine max* L. Merr.) is an important legume crop, supplying protein and oil for human and animals [1]. However, soybean growth has a lot of limitations which cause great yield losses, especially in tropical and subtropical areas where the warm, moist conditions and weathered acid soils not only favor the infection and reproduction of the pathogens [2], [3], but also easily lack of some essential nutrients, such as phosphorus (P) [4], [5]. Heavy application of chemical pesticides and fertilizers is commonly used to prevent such growth losses, but these amendments are highly cost and could cause severe environmental problems [6], [7]. Biocontrol approach should be a better alternative to solve these problems.

Red crown rot (also named black root rot) is caused by a soil-borne fungal pathogen *Cylindrocladium parasiticum* (teleomorph *Calonectria ilicicola*) [8], [9]. It was firstly found in peanut in 1965 and then in soybean in 1969 [2], [8]. Symptoms of soybean red crown rot were usually observed late in the season during or after pod set. The infected plants had a black root rot and its stalk tissue was grayish or reddish brown above soil surface for 5 to 10 cm, and leaves became cholorosis and interveinal necrosis followed by defoliation [3], [10]. Till now, more and more reports proved that red crown rot could be considered as one of the most serious soil-borne diseases for legumes around the world [3], [8], such as in China where it made soybean yield loss over 50% [2], [10].

Acid soils comprise up to 50% of the world's potentially arable land, of which syndrome causes severe yield losses in various crops, and thus significantly limit crop production worldwide [11]. In most acid soils, there are deficiencies of some essential elements, such as P, nitrogen (N), potassium (K) and some micronutrients [4], [5], [12]. Therefore, legumes like soybean grown in acid soils not only need to deal with diseases such as red crown rot but also need to face the nutrient deficiency problems.

Biocontrol approach by introducing microorganisms into soils has been proposed to suppress soil-borne diseases for improving plant health [13]. The disease suppression by biocontrol agents is regarded sustainable via harmonization of interactions among plants, pathogens, biocontrol agents and microbial communities within rhizosphere [13], [14]. Many reports found that inoculation with rhizobia and/or arbuscular mycorrhizal fungi (AMF) could promote plant growth and control fungal diseases, and thus be considered as one of the efficient biocontrol approaches [15], [16]. AMF inoculation not only helped host plant growth, but also enhanced defense system and subsequently reduced disease severity on many soil-borne pathogens [17]–[19]. Besides biological N fixation, many studies showed legumes inoculated with rhizobia could also increase host plant defense system against soil-borne diseases [20], [21]. Improvement of plant nutrition status, direct competition for invasion space, changes in root growth and morphology, as well as exudates of rhizobitoxine have been well documented as the main mechanisms underlying the inhibition of soil-borne pathogens by rhizobium and AMF inoculation [22]–[25]. Furthermore, increased transcripts of some plant pathogen defense-related (PR) gene expressions by inoculation with rhizobia and/or AMF might also be involved in plants against fungal pathogens [20], [26]. However, most of the studies described above had been conducted in greenhouse conditions and lacked of comprehensive investigations integrating field observations with physiological and molecular evidence.

Soybean could form tripartite symbiotic associations with rhizobia and AMF simultaneously for both P and N benefits [27], [28]. But until now, there is no report in whether rhizobium and/or AMF inoculation could inhibit soybean red crown rot in acid soils, and if so, what are the mechanisms underlying this inhibition? In this study, firstly, we investigated the disease incidence and index of soybean red crown rot with different P additions in acid soil field to elucidate whether the naturally inoculation of rhizobia and AMF could affect red crown rot. The field site has been growing soybean since 2000. Soybean red crown rot caused by *C. parasiticum* was observed in this field in 2006 and its disease incidence reached to 80% [10]. And then, we combined sand culture and fungal pathogen incubation experiments as well as some important PR gene expression analysis to evaluate the possible physiological and molecular mechanisms of rhizobium and AMF inoculation on soybean red crow rot inhibition.

## Results

### Field experiments

The disease incidence and index of soybean red crown rot were significantly affected by P additions in field (Table 1). Compared to HP, the plants had lower disease incidence and index at NP. Under NP conditions, disease incidence and index was decreased by 28.56% and 18.80%, 16.68% and 19.08% in 2009 and 2010, respectively.

**Table 1:** Disease incidence and index caused by *Cylindrocladium parasiticum* at different P levels in field.

P level	Disease incidence (%)		Disease index	
	2009	2010	2009	2010
NP	52.33±3.67b	51.95±1.04b	46.35±4.68b	37.32±1.74b
HP	73.25±5.32a	63.98±4.02a	55.63±3.54a	46.12±2.08a

Note: Disease incidence and index was measured as described in Materials and Methods. HP: 80 kg P_2_O_5_ ha^−1^ added as calcium superphosphate, NP: none P fertilizer added. All the data were the mean of four replicates with SE. The different letter after numbers in the same column indicated significantly different at 0.05 (*P*<0.05).

Plant growth in field was significantly affected by *C. parasiticum* infection as indicated by plant dry weight and grain yield (Table 2). Compared to the healthy plants, dry weight and grain yield of the infected plants had 39.82% and 40.09%, 39.06% and 47.80% reduction at NP and HP in 2009; 36.57% and 52.46%, 44.10% and 57.23% reduction in 2010, respectively. Interestingly, the growth of healthy plants was significantly affected by P addition, but not the infected plants. With P addition, the dry weight and grain yield of healthy plants increased 29.70% and 33.65%, 50.98% and 32.21% in 2009 and 2010, respectively. Same as plant growth, plant P and N content was significantly affected by *C. parasiticum* infection, but P and N content of the infected plants was not influenced by P level (Table S2). This suggested that the plant growth inhibition by *C. parasiticum* infection might be not directly affected by plant nutrient status.

**Table 2:** Plant growth affected by *Cylindrocladium parasiticum* infection and P level in field.

P level	Plant dry weight (g/plant)				Grain yield (g/plant)			
	Healthy Plant		Infected Plant		Healthy Plant		Infected Plant	
	2009	2010	2009	2010	2009	2010	2009	2010
NP	24.11±1.25b	24.09±1.36b	14.51±1.16a	15.28±2.39a	8.50±0.44b	9.16±0.79b	5.18±0.42a	5.12±0.85a
HP	31.27±1.84a	36.37±2.67a	17.17±1.66a	17.29±1.98a	11.36±0.63a	12.11±0.87a	5.93±0.30a	5.18±0.42a

Note: Healthy plant was not infected by *C. parasiticum*; infected plant was infected by *C. parasiticum* with severe necrosis on the subterranean stem and roots, chlorosis of leaves. HP: 80 kg P_2_O_5_ ha^−1^ added as calcium superphosphate, NP: none P fertilizer added. All the data were the mean of four replicates with SE. The same letter after numbers in the same column indicated not significantly different at 0.05 (*P*<0.05).

**Figure 1:**
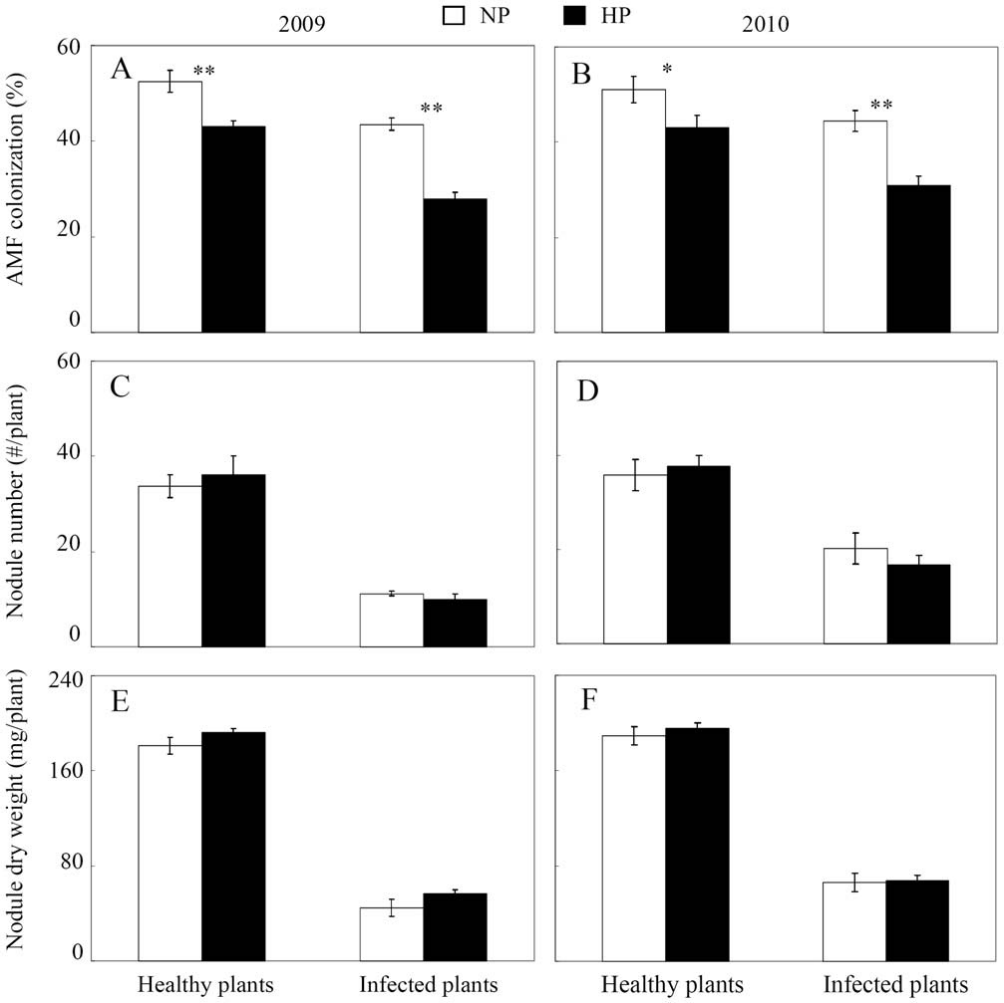
AMF colonization and nodulation affected by *C. parasiticum* and P level in field. A) and B) AMF colonization; C) and D) nodule number; E) and F) nodule dry weight. HP: 80 kg P_2_O_5_ ha^−1^ added as calcium superphosphate; NP: none P fertilizer added. Healthy plants were not infected by *C. parasiticum*, infected plants were infected by *C. parasiticum* with severe necrosis on the subterranean stem and roots, chlorosis of leaves. Each bar represented the mean of four replicates with standard error. * and ** stood the significant difference between two P levels analyzed by one-way ANOVA. *: significant at 0.05 (P<0.05); **: significant at 0.05 (P<0.01).

Mycorrhization and nodulation of roots were significantly inhibited by *C. parasiticum* infection as indicated by AMF colonization rate, nodule number and nodule dry weight in field, respectively (Figure 1). Compared to the healthy plants, the infected plants had 17.78%, 66.67% and 75.14%; 35.75%, 72.22% and 70.31% decrease of AMF colonization rate, nodule number and dry weight at NP and HP in 2009, 15.80%, 43.36% and 65.08%; 30.25%, 55.63% and 60.23% decrease in 2010, respectively. Compared to mycorrhization, nodulation showed more severe inhibition by *C. parasiticum* infection. But AMF colonization rate was more negatively affected by P addition, especially for the healthy plants. The AMF colonization rate of the healthy plants decreased 17.02% and 13.04% at HP in 2009 and 2010 compared to NP treatment, respectively. This indicated that there were complicate interactions among mycorrhization, nodulation, *C. parasiticum* infection and P status in the field of acid soils.

### Sand culture experiment

In order to understand the relationships among *C. parasiticum* pathogen infection, AMF colonization and nodulation as well as plant nutrient status, a sand culture experiment was further conducted. As expected, plant growth and nutrient status were negatively affected by pathogen infection but promoted by rhizobium and AMF inoculation under both LP and HP conditions as indicated by plant dry weight, N and P content (Figure S1). Furthermore, the suppression of plant growth and nutrient status derived from pathogen infection were better rescued by co-inoculation with rhizobia and AMF. Interestingly, co-inoculation with rhizobia and AMF further increased AMF colonization rate compared to AMF inoculation alone, and *vice versa*. The P availability imposed positive effects on nodulation but negative effects on mycorrhization as indicated by nodule number and nodule dry weight as well as AMF colonization rate, respectively (Figure S2).

**Figure 2:**
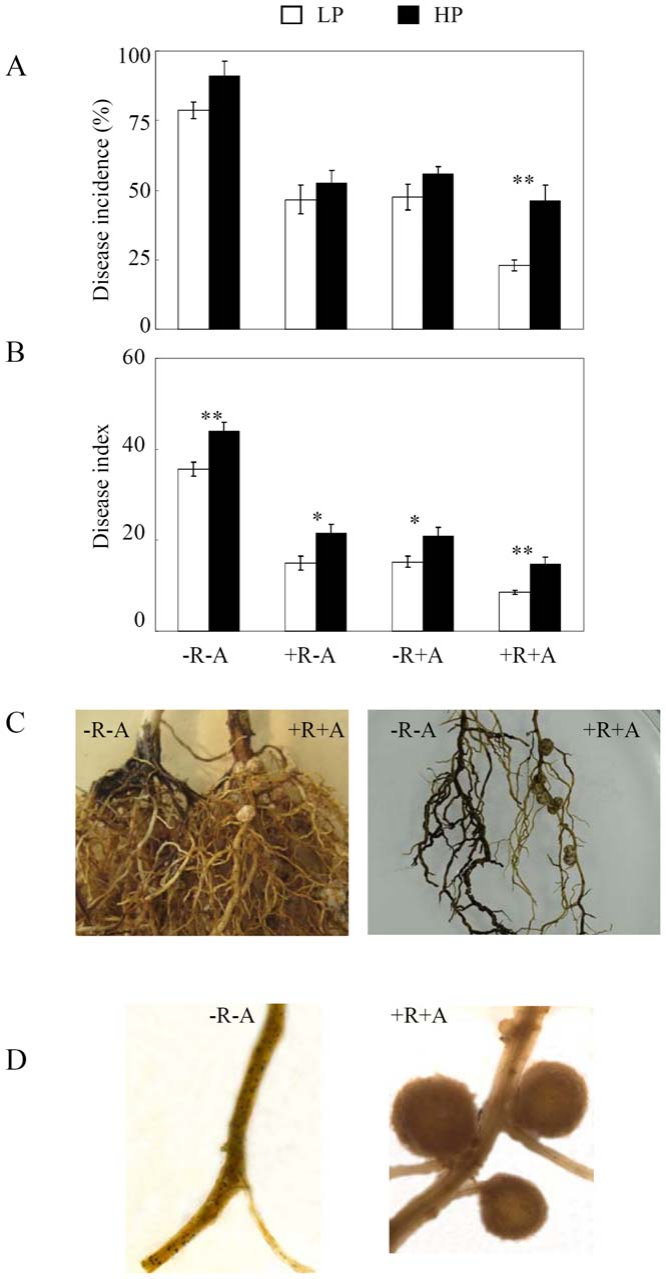
Disease incidence and index of soybean red crown rot affected by *C. parasiticum* infection, rhizobia and AMF inoculation as well as P level in the sand culture experiment. A) disease incidence; B) disease index; C) pictures of *C. parasiticum* infection, rhizobia and AMF inoculation on tap root (left) and lateral roots (right); D) microsclerotia in the roots. LP, 15 µmol P added as KH_2_PHO_4_; HP, 500 µmol P added as KH_2_PHO_4_. Disease incidence and index was measured as described in Materials and Methods. All the roots were inoculated with *C. parasiticum* (see Materials and Methods for details). −R−A: roots without AMF and rhizobia inoculation; +R−A: roots inoculated by rhizobia; −R+A: roots inoculated by AMF; +R+A: roots inoculated by rhizobia and AMF. Each bar represented the mean of eight replicates with standard error. * and ** stood the significant difference between two P levels analyzed by one-way ANOVA. *: significant at 0.05 (P<0.05); **: significant at 0.05 (P<0.01).

We found that the disease incidence and index of soybean red crown rot were dramatically decreased after rhizobium and/or AMF inoculation in sand culture (Figure 2), indicating that symbiotic inoculation could not only lower the pathogen colonization but also reduce the root rot severity. Plants co-inoculated with rhizobia and AMF always had the lowest disease incidence and index, especially at low P level. Furthermore, the disease incidence and index decreased from 78.65% and 35.60 without inoculation to about 45% and 15, with rhizobial or AMF inoculation, and further decreased to 22.93% and 8.64 with co-inoculation at low P level, showing significant interactions between rhizobium and AMF inoculation on pathogen control. Interestingly, we found that both tap root and lateral roots where AMF and rhizobia colonized had less microsclerotia (Figure 2C and 2D), implying that space competition might be occurred among AMF, rhizobium and *C. parasiticum* infection.

**Figure 3:**
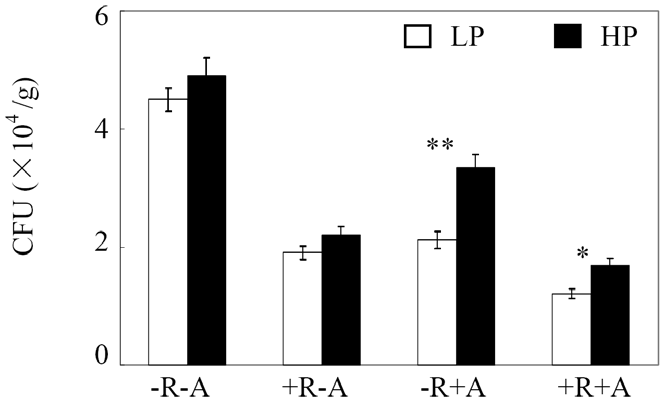
*C. parasiticum* pathogen growth affected by rhizobia and AMF inoculation as well as P level in the sand culture experiment. LP, 15 µmol P added as KH_2_PHO_4_; HP, 500 µmol P added as KH_2_PHO_4_. All the roots were inoculated with *C. parasiticum* (see Materials and Methods for details). CFU: colony forming units per gram root sample. −R−A: roots without AMF and rhizobia inoculation; +R−A: roots inoculated by rhizobia; −R+A: roots inoculated by AMF; +R+A: roots inoculated by rhizobia and AMF. Each bar represented the mean of four replicates with standard error. * and ** stood the significant difference between two P levels analyzed by one-way ANOVA. *: significant at 0.05 (P<0.05); **: significant at 0.05 (P<0.01).

To further investigate the effects of symbiotic inoculation on *C. parasiticum* pathogen colony growth in roots, we measured CFU and found that the CFU caused by *C. parasiticum* was significantly inhibited by rhizobium and AMF inoculation (Figure 3). The CFU amounts were 57.78%, 52.89% and 73.33% lower under +R−A, −R+A and +R+A treatments compared to −R−A at LP level, respectively. P addition only significantly enhanced the *C. parasiticum* pathogen growth when inoculated with AMF as indicated by 58.02% and 40.42% increase of CFU under −R+A and +R+A treatments at HP, respectively.

**Figure 4:**
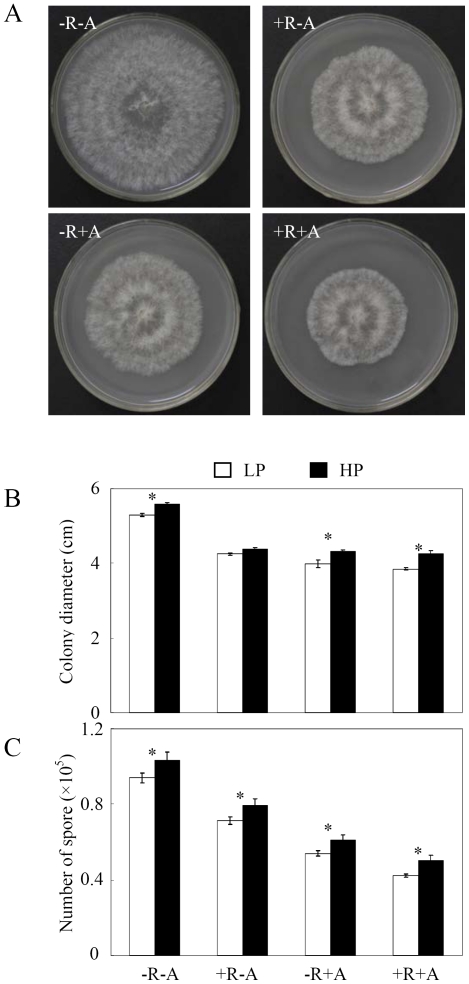
Mycelial radial growth and sporulation of *C. parasiticum* affected by the exudates from the roots with rhizobia and/or AMF inoculation as well as P level. LP, 15 µmol P added as KH_2_PHO_4_; HP, 500 µmol P added as KH_2_PHO_4_. a) pictures of pathogen colony at LP; b) colony diameter of pathogen; c) number of spores. −R−A: roots without AMF and rhizobia inoculation; +R−A: roots inoculated by rhizobia; −R+A: roots inoculated by AMF; +R+A: roots inoculated by rhizobia and AMF. Each bar represented the mean of four replicates with standard error. * and ** stood the significant difference between two P levels analyzed by one-way ANOVA. *: significant at 0.05 (P<0.05); **: significant at 0.05 (P<0.01).

The growth of *C. parasiticum* pathogen was significantly inhibited by the exudates from the roots inoculated with rhizobia and/or AMF under different P conditions as indicated by pathogen colony diameter and sporulation, especially at LP (Figure 4). In comparison with control, the addition of 2 mL root exudates from plants with rhizobium and/or AMF inoculation dramatically inhibited pathogen growth (Figure 4A). Under LP conditions, the pathogen colony diameter and sporulation of *C. parasiticum* decreased 27.36% and 55.31% after co-inoculation with rhizobia and AMF compared to control, showing the strong synergistic interactions between rhizobium and AMF inoculation on inhibiting *C. parasiticum* growth. This suggested that the symbiotic inoculation induced/enhanced the ability of host plants to secrete some or more defense-compounds to control pathogen infection. Furthermore, there were four phenolic acids detected in the root exudates (Table 3). Among them, roots from the −R−A treatment only released gallic acid, and the concentration was enhanced by rhizobia and/or AMF inoculation. Interestingly, the other three detected phenolic acids, including ferulic acid, cinnamic acid and salicylic acid were only secreted from the roots inoculated with rhizobia and/or AMF at both P levels, indicating that the changes of phenolic acids in root exudates might be the main compounds controlling the pathogen infection in current conditions.

**Table 3:** The concentration of phenolic acids (µg·g^−1^ root DW) in root exudates from different treatments at flowering stage detected by HPLC.

Treatment		Ferulic acid	Gallic acid	Cinnamic acid	Salicylic acid
LP	−R−A	-	1.03±0.13	-	-
	+R−A	1.78±0.16	0.79±0.08	-	1.67±0.17
	−R+A	2.11±0.18	-	0.87±0.06	-
	+R+A	2.31±0.21	1.32±0.12	1.33±0.11	1.71±0.15
HP	−R−A	-	0.37±0.04	-	-
	+R−A	1.34±0.12	1.84±0.17	-	2.11±0.22
	−R+A	2.05±0.22	-	0.76±0.06	-
	+R+A	1.83±0.15	1.51±0.13	0.83±0.09	1.86±0.19

Note: “-” refers to not detectable; LP, 15 µmol P added as KH_2_PHO_4_; HP, 500 µmol P added as KH_2_PHO_4_. −R−A: roots without AMF and rhizobia inoculation; +R−A: roots inoculated by rhizobia; −R+A: roots inoculated by AMF; +R+A: roots inoculated by rhizobia and AMF. All the data were the mean of four replicates with SE.

### Plant defense-related gene expression analysis

**Figure 5:**
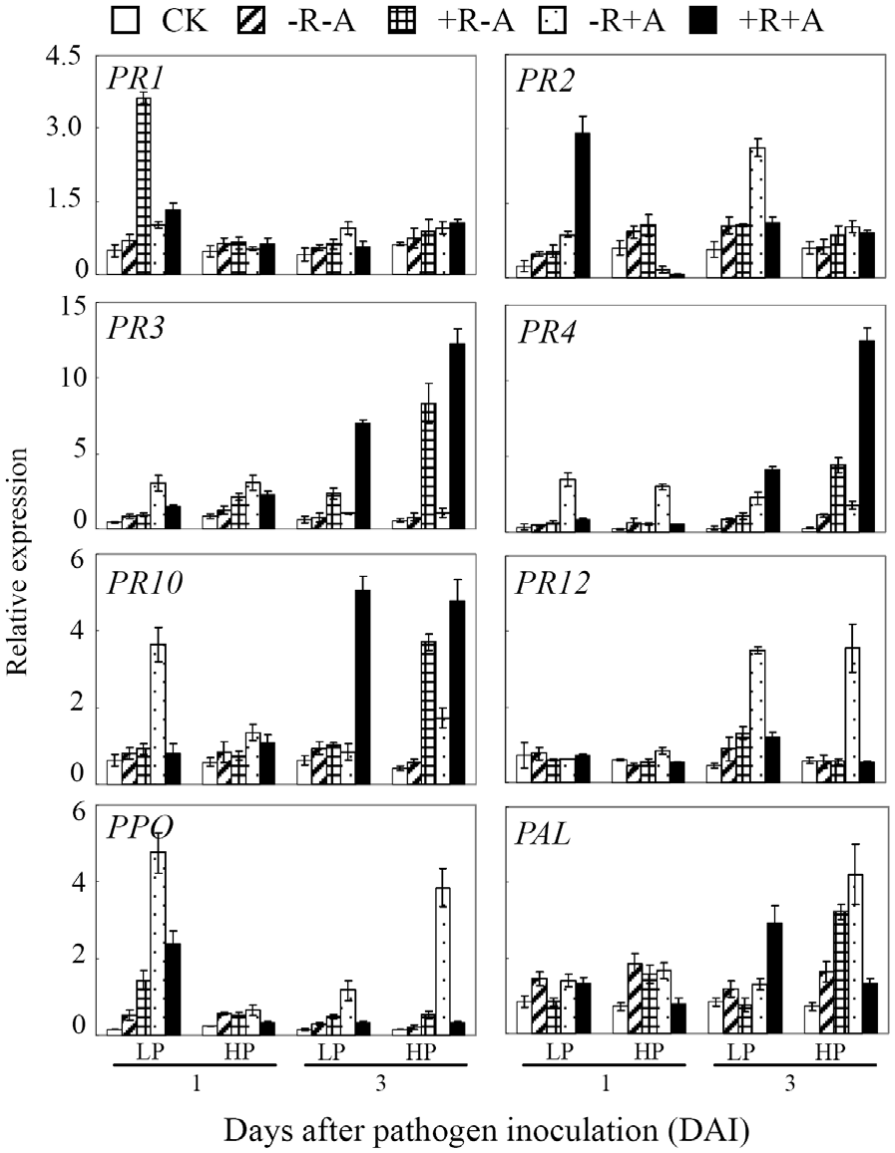
Expression changes of eight defense-related genes in roots of soybean in response to *C. parasiticum* infection and rhizobia and/or AMF inoculation as well as P level. LP, 15 µmol P added as KH_2_PHO_4_; HP, 500 µmol P added as KH_2_PHO_4_. Except CK, all the roots were inoculated with *C. parasiticum* (see Materials and Methods for details). CK: roots without *C. parasiticum*, AMF and rhizobia inoculation; −R−A: roots without AMF and rhizobia inoculation; +R−A: roots inoculated by rhizobia; −R+A: roots inoculated by AMF; +R+A: roots inoculated by rhizobia and AMF. Each bar represented the mean of three biological replicates with standard error.

Quantitative RT-PCR was used to assay expression patterns of 8 pathogen defense-related (PR) genes in both roots and leaves. Results showed that the transcripts of the most tested genes in roots were significantly increased by rhizobium and/or AMF inoculation, which was partly dependent on P availability (Figure 5). Except that significant responses of *PR12* to rhizobium and/or AMF inoculation were only observed at 3 DAI (Day After Inoculation), transcripts of the other 7 genes were significantly increased at both 1 and 3 DAI. Furthermore, the genes responded differentially to rhizobium and/or AMF inoculation. Among them, transcription levels of *PR2*, *PR3*, *PR4* and *PR10* reached the highest level with co-inoculation of rhizobium and AMF. However, the most transcripts of *PR12*, *PPO* and *PAL* were observed at AMF inoculation. Interestingly, transcripts of some genes with rhizobium and/or AMF inoculation were up-regulated by P application, especially at 3 DAI. For example, *PR3*, *PR4*, *PR10* and *PAL* with rhizobium inoculation, *PR10*, *PPO* and *PAL* at AMF inoculation, *PR3* and *PR4* at rhizobium and AMF co-inoculation exhibited higher expression levels with P application at 3 DAI.

Similar responses of the genes to rhizobium and/or AMF inoculation were also observed in leaves (Figure S3). However, the highest responses of 6 genes to rhizobium inoculation were observed, including *PR2*, *PR3*, *PR4*, *PR10*, *PR12* and *PPO*. The other 2 genes responded mostly to AMF inoculation, including *PR1* and *PAL*. Furthermore, P availability was also involved in regulating expression patterns of the genes. Except *PAL*, transcripts of all the other genes were increased by P application at rhizobium inoculation at 1DAI. At 3 DAI, P application also resulted in increasing transcripts of the genes, including *PR1*, *PR2* and *PR3* at rhizobium inoculation, *PR1*, *PR2*, *PR4* and *PAL* at AMF inoculation.

## Discussion

The severe yield losses caused by red crown rot have been estimated to be as high as 50% [2], [10]. In earlier reports, the research of the relationship between symbionts and pathogens was mostly conducted in greenhouse or laboratory under better controlled conditions [29], [30], but no report has been carried out in both field and greenhouse. Therefore, our study should be the first comprehensive research combined field, sand culture as well as pathogen incubation experiments, from phenotype to physiological and molecular analysis to elucidate the underlying relationships and mechanisms among symbionts and soybean red crown rot as well as P application in acidic red soils.

In this study, we found that inoculation with rhizobia or AMF could inhibit soybean red crown rot happening and development, and co-inoculation get more effective results than using a single microsymbiont in sand culture (Figure 2). From the field experiment, we also found that mycorrhization and nodulation of roots were significantly inhibited by *C. parasiticum* infection (Figure 1), implying that under the condition of simultaneously naturally inoculated by indigenous rhizobia, AMF and pathogen in field, there might be vital competition and antagonism effects occurred. Firstly, rhizosphere colonization was important not only as the first step in pathogenesis of soil borne microorganisms but also was crucial in the application of microorganisms for beneficial purposes [31]. Rhizobia and AMF should be able to compete with pathogens through efficiently colonizing the rhizosphere of the plants [22], [32]. Secondly, rhizobia and AMF might compete for the colonizing sites of plant roots with pathogens, and thus protect plants out of pathogen infection as the roles of most biocontrol agents [32], [33]. This competition effect could be supported by our results from sand culture that tap root and lateral roots colonized by AMF and rhizobia had less pathogen microsclerotia (Figure 2C). Furthermore, preventing growth and proliferation of phytopathogens through the toxic metabolites produced by rhizobium and AMF is another well accepted mechanism of biocontrol agents [34]–[36]. We found that the root exudates from nodulated and/or AMF colonized roots directly interfered with the growth and proliferation of pathogens *in vitro* (Figure 4), which suggested that more antibiotics might be produced by plants when inoculated with rhizobia and/or AMF, and thus reduce pathogen growth and proliferation. In our study, we found that the phenolic acids in the root exudates were coincidently enhanced by rhizobia and/or AMF inoculation (Table 3). Therefore, we speculated that the phenolic acids in the root exudates might be the critical biological control substances for *C. parasiticum* infection in soybean. The detailed mechanisms are needed to be further elucidated.

Moreover, it has been well documented that inoculation with rhizobia and/or AMF had potential impacts to the disease occurrence and development [37], [38], which probably due to the increase of plant growth and nutrient status after roots colonized by rhizobia and AMF [39], [40]. However, in our study, we found that even P fertilizer addition in both field and sand culture experiments enhanced plant growth, N and P nutrition (Table 2 and Table S2), but also increased the disease incidence and index of red crown rot no matter inoculation with rhizobia and AMF or not (Table 1 and Figure 2), indicating that direct promotion of plant growth and nutrient status is not the mechanism of the inhibition effect of rhizobium and AMF inoculation on *C. parasiticum* infection. The similar results are also found in many garden plants that P application increase the severity of disease caused by *Sclerotinia sclerotiorum* [41], [42]. Furthermore, high P availability has been demonstrated to play opposite roles in nodulation and mycorrhization as indicated by enhanced nodulation but suppressed mycorrhization with increasing P availability [28]. We also found that P addition significantly decreased AMF colonization rate in both field and sand culture (Figure 1 and Figure S2), implying that the increased disease incidence and index of *C. parasiticum* could be partly due to the decrease of AMF colonization rate. This is also supported by the results of root pathogen colony growth from sand culture. The CFU only enhanced by high P when inoculated with AMF (Figure 3). Therefore, for using rhizobia and AMF inoculation to control soybean red crown rot in acid soils, proper P application should be further considered.

In addition, activation of specific plant defense mechanisms in response to symbiont colonization is also an obvious basis for the protective behavior of rhizobia and AMF [20], [43], [44]. Compounds associated with plant defense included, but not limited to, phytoalexins, chitinases, β-1, 3-glucanases, pathogenesis related proteins, callose and phenolics [17], [45], [46]. In legumes, a number of genes encoding pathogenesis related proteins have been showed to be locally and systemically up-regulated after inoculation with pathogens [47]–[49]. Furthermore, the increase of some specific plant defense-related (PR) gene expressions to rhizobium and/or AMF colonization has been reported to against fungal pathogens [24], [26]. For example, inoculation with rhizobium could induce the PR genes involved in phytoalexin synthesis and made plants primed for against the fungal pathogen, and then reduce the severity of disease [26]. Phenolics were observed in rhizobium treated seedlings against the pathogen infection, which might be controlled by *PAL* [50]. Mycorrhization also amplified the accumulation of the PR genes and phytoalexins synthesis in fungal pathogen infected [51]–[53]. Our results through q-PCR analysis found that inoculation with rhizobia and/or AMF could significantly increase the expression of most selected PR genes in both roots and leaves at 1 and 3 DAI (Figure 5), especially under co-inoculation conditions. This indicated that inoculation with rhizobia and/or AMF in our study activated both local and systemic defense systems in a short- and long-term way in soybean plants. The similar elicitation via AMF and rhizobia symbiosis to activate specific defense reactions and predispose an early response when attacked by a root pathogen has been found in legumes [17], [53]. Interestingly, we also found that inoculation with rhizobia up-regulated more PR genes in roots, but AMF up-regulated more in leaves (Figure 5 and Figure S3), suggesting that the plant responds to rhizobium and AMF inoculation through different defense systems. Plants prefer to activate local defense systems to rhizobium inoculation, but systemic defense systems to AMF inoculation. Co-inoculation could activate both defense systems and result in better pathogen inhibition.

Take together, our study firstly showed that inoculation with rhizobia and AMF could decrease soybean red crown rot occurrence and development, through direct evidence in inhibition of pathogen growth and reproduction, and enhancement of some PR gene expressions. The study indicated that the biocontrol method and nutrient management, like inoculation with rhizobia and AMF and optimal P fertilization, could be considered as an efficient method to control soybean red crown rot in acid soils, and therefore apply practical measure for sustainable soybean production.

## Materials and Methods

### Field experiments

Soybean genotype HN112 was grown on acidic red soils at Boluo (E114.28°, N23.18°) experimental site of South China Agricultural University in Guangdong Province of China in 2009 and 2010. Basic soil chemical characteristics were as follows: pH, 5.37; organic matter, 17.63 g kg^−1^; available P (Bray I method), 15.68 mg P kg^−1^; available nitrogen, 86.64 mg N kg^−1^; available potassium, 75.28 mg K kg^−1^.

There were two P levels, including high P (80 kg P_2_O_5_ ha^−1^ added as calcium superphosphate, HP) and low P (none P fertilizer added, NP). The P fertilizer was applied to the topsoil by spread application. Each treatment had four replicates in a randomized complete block design with 8 plots in total. Each plot had an area of 18 m^2^ and the planting density was 21 plants m^−2^ (40 cm between rows, 15 cm between plants). The experimental field was managed following local farmer's practices. Seventy days after planting, 100 plants were examined in each plot for the damage caused by red crown rot. Two representative healthy and infected plants were harvested from each replicate for further analysis. Plant dry weight, grain yield, plant N and P content, nodule number, nodule dry weight and AMF colonization rate were measured after harvest. Plants and nodules were dried at 105°C for 30 min, and then kept at 75°C till completely dry to determine dry weight. Total N and P content were measured using the semi-micro Kjedahl procedure with a N analyzer (Kjedahl 2300; FOSS, Hoganas, Sweden) and phosphorus–molybdate blue color reaction, respectively [54].

Disease incidence was defined as the percentage of diseased subterranean stems. Disease severity was recorded for each plant on a 0–5 scale according to Nishi [55]: i.e., 0 = no visible symptoms; 1 = small necrotic lesions on the subterranean stem; 2 = necrotic lesions extending around the subterranean stem, 3 = necrotic lesion on the subterranean stem extending to the groundline; 4 = severe necrosis on the subterranean stem and roots, leaf chlorosis; 5 = plant dead. Disease index was summarized within each plot as {[(n_1_×1)+(n_2_×2)+(n_3_×3)+…+(n_N_×N)]/[N×(n_1_+n_2_+n_3_…+n_N_)]}×100, where n_1_…n_N_ was the number of subterranean stems in each of the respective disease categories, N was the highest scoring of the disease [56]. Fifty roots of each plot with 1 cm length were sampled for AMF assay. The roots were cleared by 10% KOH for 7 days, and stained with 5% ink-vinegar solution according to the procedure of Vierheilig [57]. Total AMF colonization rate of roots was determined as the percentage of root length colonized by AMF using the intersection method.

### Sand culture experiment

The sand culture experiment was conducted in the greenhouse of Root Biology Center at South China Agricultural University using soybean genotype HN112 as plant materials. Each pot consisted of 2 kg of silica sand and 6 soybean plants. The silica sand was sterilized by autoclave on two consecutive days for 40 min at 121°C. There were 3 factors including P level, pathogen infection and symbiotic inoculation, and thus 16 treatments in total. Two P levels were HP (500 µM P added as KH_2_PO_4_) and LP (15 µM P added as KH_2_PO_4_). Pathogen infection was treated as added *C. parasiticum* spore suspensions or sterilized spore suspensions as control. For symbiotic inoculation, there were 4 inoculation levels: rhizobium inoculation alone (+R−A, 20 mL rhizobia liquid per pot), AMF inoculation alone (−R+A, 100 g AMF inoculants per pot), co-inoculation with rhizobium and AMF (+R+A, 20 mL rhizobia liquid and 100 g AMF inoculants per pot) and none inoculation as control (−R−A, 100 g sterilized AMF inoculants and 20 mL sterilized rhizobia liquid per pot). Inoculants with rhizobium and/or AMF was mixed with silica sand before planting. There were 8 replicates for each treatment in this study.

The pathogen used in this study was *C. parasiticum* (GenBank Accession No. GU073284) isolated from the infected soybean roots in Boluo field site [10]. Spore suspensions of plant pathogens were obtained from 14-day-old V8-juice media which were collected by adding 10 mL of sterile water to each Petri dish and rubbing the surface with a sterile L-shaped spreader. The suspension was subsequently filtered through 3-layers of cheesecloth. The spore concentration was determined using a hemacytometer and adjusted to 1×10^5^ spores per mL.

The rhizobium inoculant was made by *Bradyrhizobium sp.* BXYD3 with MPN value of 1×10^9^ rhizobia per mL of liquid [58]. The original AMF inoculum was *Glomus mosseae* from maize (*Zea mays* L.) with MPN value as 300 progagules per gram of soils. The AMF inoculants were a mixture of the infected maize roots, spores and mycelium [58]. AMF and rhizobium inoculants were mixed into sand before planting. Thirty days after planting, plant stem base was infected with 20 mL *C. parasiticum* spore suspensions each pot. Plants were irrigated once every day with modified 1/2 strength Hoagland nutrient solution with either of the two P additions as mentioned above [12]. Sixty days after planting, plants were investigated the damage caused by red crown rot as described above, and then harvested. Plant dry weight, N and P content, nodule number, nodule dry weight, AMF colonization were measured as mentioned above. The microsclerotia in the roots were observed under a Stereo microscope (Leica, M165C, Germany).

### Determination of root infection by *Cylindrocladium parasiticum*

In order to determine the root infection by *C. parasiticum*, root colony forming unit (CFU) was measured 30 days after pathogen inoculation in sand, randomly selected soybean roots were cut into 1 cm segments and washed thoroughly and surface-sterilized with 0.5% (v/v) NaClO for 3 min, rinsed three times in sterilized water, and then blotted on sterilized filter paper. About 1 g root segments were ground in 50 mL of sterile deionized water using a blender at high speed for 1 min [59]. The homogenized root suspension was diluted 10 folds with sterile deionized water. 100 µL dilution was placed and evenly spread on Rose Bengal Medium with a sterile glass rod for 4 plates. There were 4 replicates and thus 16 plates for each treatment. All plates were incubated at 28°C in dark for 4 days. Colonies of *C. parasiticum* were identified and counted on each plate to determine the CFUs per gram of roots for each treatment.

Root exudates of soybean seedlings infected by *C. parasiticum* from the sand culture experiments were collected at flowering stage, and then added to V8-juice medium during *C. parasiticum* incubation for studying the effects of root exudates on mycelial radial growth and sporulation of *C. parasiticum*. Roots were gently taken out of the sand and washed with deionized water. The cleaned roots were submerged in a plastic cup containing 500 mL of 0.5 µM CaCl_2_ to collect exudates for 6 hours. Root exudates were filtered by 0.45 µm Millipore membrane and stored at −20°C. During collection, each cup containing 3 seedlings were covered by a black plastic lid to avoid contamination and light. The grown diameter and sporulation of pathogen was conducted by adding 2 mL root exudates to V8-juice medium before it was solidified to give a total volume of 20 mL per Petri dish. Plates were incubated at 28°C in dark, the colony diameter and sporulation of *C. parasiticum* was determined on 5 and 14 days after incubation. For phenolic acid detection, the root exudates were firstly evaporated in a rotary evaporator (Free Zone Freeze Dry Systems, LABCONCO, USA), and then analyzed by HPLC (Agilent 1200, USA) according to Banwart [60].

### Plant defense-related gene expression analysis

In order to study the responses of plant defense-related genes to *C. parasiticum* infection, AMF and rhizobia inoculation, total RNA from leaves and roots of soybean plants were separately extracted according to Guo [61]. Thirty-day soybean seedlings grown in sand at both low P and high P levels were used in this study. Leaves and roots for each treatment were randomly harvested at 0, 1, 2, 3 days after pathogen inoculation, respectively. Leaves and roots were washed with tap water and immediately frozen in liquid nitrogen. Total RNA was extracted and isolated according to the method of Plant RNA Mini Kit as described by the manufacturer (Omega, GA, USA). First strand cDNA was synthesized from the 1 µg of total RNA using ImProm-II™ Reverse transcription system according to the manufacturer's instructions (Promega, Madison, USA). All the reactions were done on a Rotor-Gene 3000 (Corbett Research, Australia). The soybean housekeeping gene *EF1-α* (Accession number: X56856) was used as endogenous control to normalize the samples. Eight plant defense-related genes were selected to verify their expression patterns by quantitative real time-PCR (qRT-PCR). The specific primer sequences and putative functions of the tested plant defense-related genes were listed in Table S1.

### Data Analysis

All the data in the experiments were statistically analyzed by ANOVA using Excel 2003 software (Microsoft Corporation, 1985–2003) and SAS 8.1 (SAS Inc., Cary, NC, USA).

## Supporting Information

Figure S1 **Plant dry weight, P and N content affected by** ***C. parasiticum*** **infection, rhizobia and AMF inoculation as well as P level in sand culture experiment.** A) plant dry weight; B) plant P content; C) plant N content. LP, 15 µmol P added as KH_2_PHO_4_; HP, 500 µmol P added as KH_2_PHO_4_. Besides CK, all the roots were inoculated with *C. parasiticum* (see Materials and Methods for details). CK: roots without *C. parasiticum*, AMF and rhizobia inoculation; −R−A: roots without AMF and rhizobia inoculation, +R−A: roots inoculated by rhizobia, −R+A: roots inoculated by AMF, +R+A: roots inoculated by rhizobia and AMF. Each bar represents the mean of four replicates with standard error.
(TIF)

Figure S2 **AMF colonization, nodule number and dry weight affected by** ***C. parasiticum*** **, rhizobia and AMF inoculation as well as P level in sand culture experiment.** A) nodule number; B) nodule dry weight; C) AMF colonization. LP: 15 µmol P added as KH_2_PHO_4_; HP: 500 µmol P added as KH_2_PHO_4_. Besides CK, all the roots were inoculated with *C. parasiticum* (see Materials and Methods for details). CK: roots without *C. parasiticum*, AMF and rhizobia inoculation; −R−A: roots without AMF and rhizobia inoculation, +R−A: roots inoculated by rhizobia, −R+A: roots inoculated by AMF, +R+A: roots inoculated by rhizobia and AMF. Each bar represents the mean of four replicates with standard error.
(TIF)

Figure S3 **Expression changes of eight defense-related genes in leaves of soybean in response to** ***C. parasiticum*** **infection and rhizobia and/or AMF inoculation as well as P level.** LP, 15 µmol P added as KH_2_PHO_4_; HP, 500 µmol P added as KH_2_PHO_4_. Except CK, all the roots were inoculated with *C. parasiticum* (see Materials and Methods for details). CK: roots without *C. parasiticum*, AMF and rhizobia inoculation; −R−A: roots without AMF and rhizobia inoculation; +R−A: roots inoculated by rhizobia; −R+A: roots inoculated by AMF; +R+A: roots inoculated by rhizobia and AMF. Each bar represented the mean of three biological replicates with standard error.
(TIF)

Table S1 **Real Time PCR primers designed for this study.**
(DOC)

Table S2 **Plant N and P content affected by** ***C. parasiticum*** **infection and P level in field.**
(DOC)
