# Supplementary figures and images for: Co-Inoculation with Rhizobia and AMF Inhibited Soybean Red Crown Rot: From Field Study to Plant Defense-Related Gene Expression Analysis
Source: PLoS One. 2012 Mar 19;7(3):e33977. doi: 10.1371/journal.pone.0033977 (PMC3307780; doi:10.1371/journal.pone.0033977)

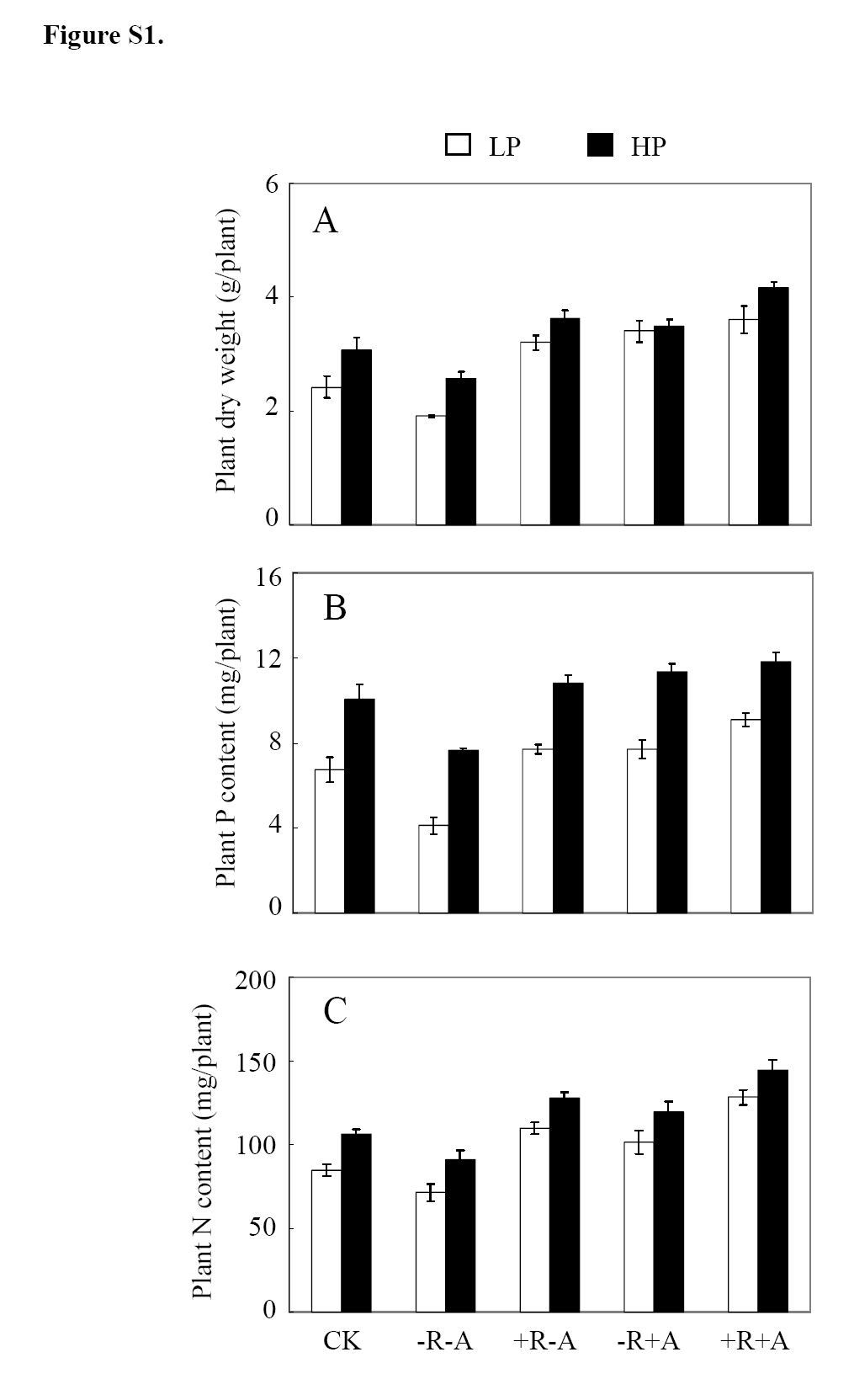

Supplement: Figure S1 — Plant dry weight, P and N content affected by C. parasiticum infection, rhizobia and AMF inoculation as well as P level in sand culture experiment. A) plant dry weight; B) plant P content; C) plant N content. LP, 15 µmol P added as KH2PHO4; HP, 500 µmol P added as KH2PHO4. Besides CK, all the roots were inoculated with C. parasiticum (see Materials and Methods for details). CK: roots without C. parasiticum, AMF and rhizobia inoculation; −R−A: roots without AMF and rhizobia inoculation, +R−A: roots inoculated by rhizobia, −R+A: roots inoculated by AMF, +R+A: roots inoculated by rhizobia and AMF. Each bar represents the mean of four replicates with standard error. (TIF) [file pone.0033977.s001.tif]

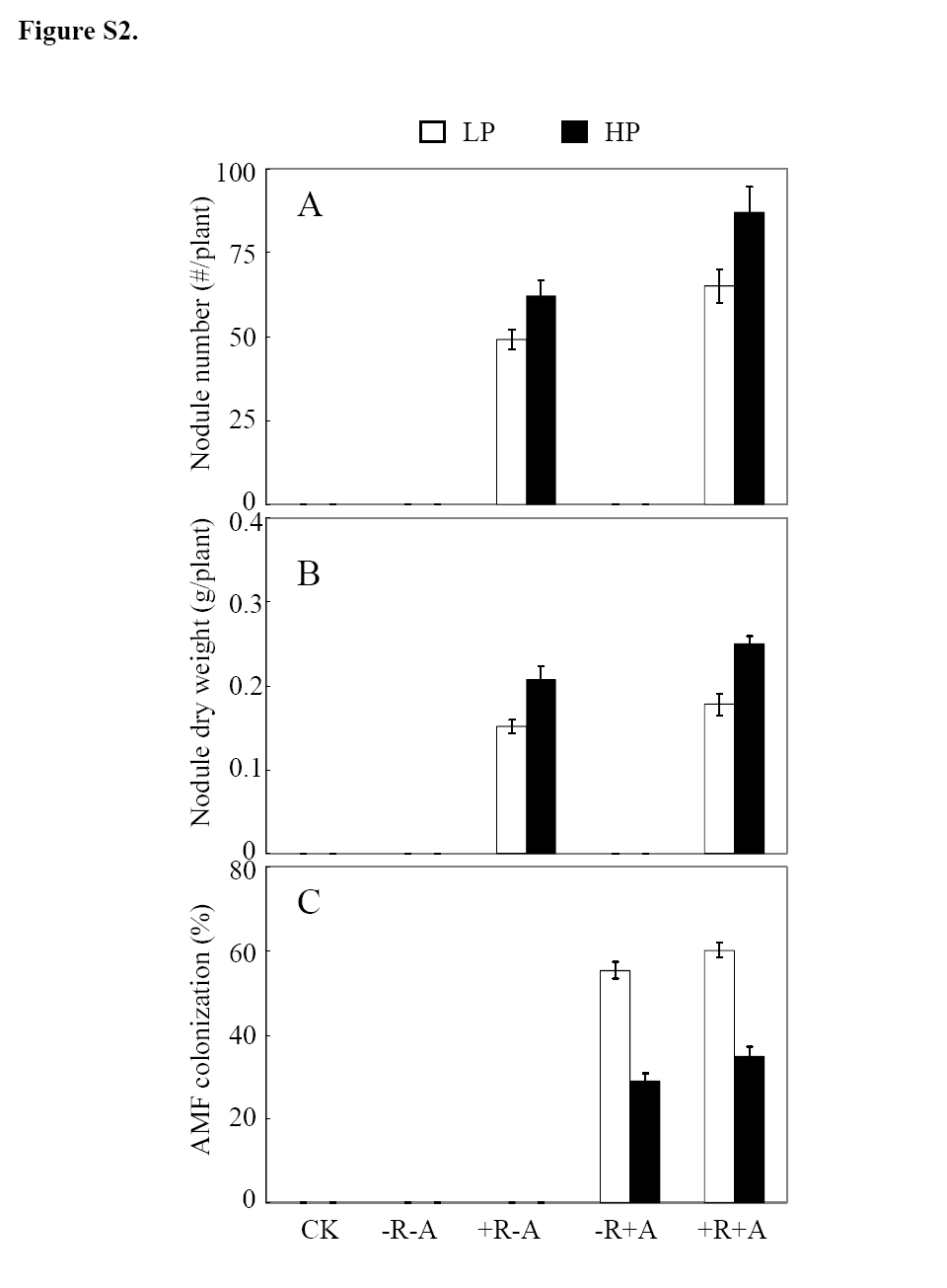

Supplement: Figure S2 — AMF colonization, nodule number and dry weight affected by C. parasiticum , rhizobia and AMF inoculation as well as P level in sand culture experiment. A) nodule number; B) nodule dry weight; C) AMF colonization. LP: 15 µmol P added as KH2PHO4; HP: 500 µmol P added as KH2PHO4. Besides CK, all the roots were inoculated with C. parasiticum (see Materials and Methods for details). CK: roots without C. parasiticum, AMF and rhizobia inoculation; −R−A: roots without AMF and rhizobia inoculation, +R−A: roots inoculated by rhizobia, −R+A: roots inoculated by AMF, +R+A: roots inoculated by rhizobia and AMF. Each bar represents the mean of four replicates with standard error. (TIF) [file pone.0033977.s002.tif]

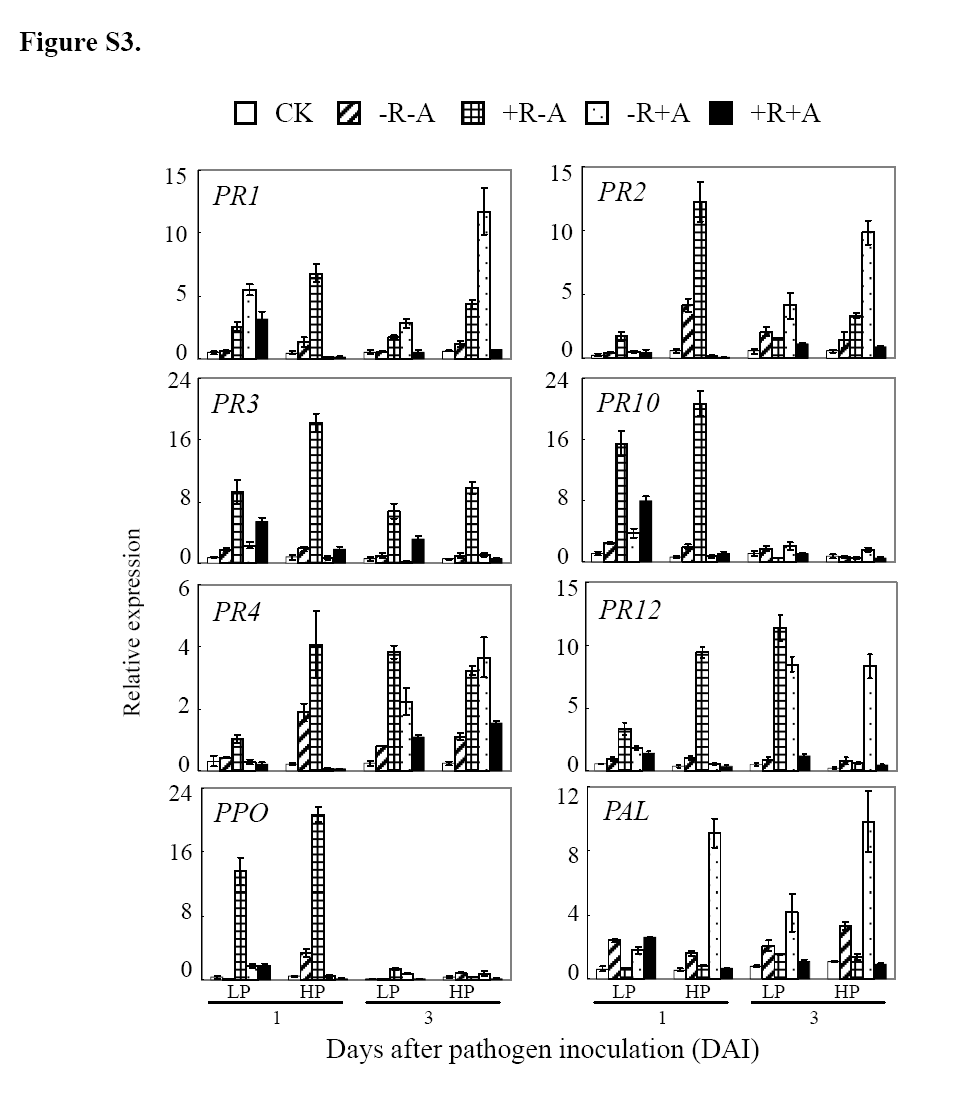

Supplement: Figure S3 — Expression changes of eight defense-related genes in leaves of soybean in response to C. parasiticum infection and rhizobia and/or AMF inoculation as well as P level. LP, 15 µmol P added as KH2PHO4; HP, 500 µmol P added as KH2PHO4. Except CK, all the roots were inoculated with C. parasiticum (see Materials and Methods for details). CK: roots without C. parasiticum, AMF and rhizobia inoculation; −R−A: roots without AMF and rhizobia inoculation; +R−A: roots inoculated by rhizobia; −R+A: roots inoculated by AMF; +R+A: roots inoculated by rhizobia and AMF. Each bar represented the mean of three biological replicates with standard error. (TIF) [file pone.0033977.s003.tif]
