# Supplementary material for: Co-Inoculation with Rhizobia and AMF Inhibited Soybean Red Crown Rot: From Field Study to Plant Defense-Related Gene Expression Analysis
Source: PLoS One. 2012 Mar 19;7(3):e33977. doi: 10.1371/journal.pone.0033977 (PMC3307780; doi:10.1371/journal.pone.0033977)
Supplement: Table S1 — Real Time PCR primers designed for this study. (DOC) [file pone.0033977.s004.doc]

Table S1. Real Time PCR primers designed for this study

| Gene | Forward/reverse primers | Target sequence^a^ | Ta (℃)^b^ | Amplicon (bp) | Description | Reference |
| --- | --- | --- | --- | --- | --- | --- |
| *PR1* | TGATGTTGCCTACGCTCAAG | AF136636 | 61 | 137 | PR1a precursor | [46] |
|  | AAGCAGCAACCGTATCATCC |  |  |  |  |  |
| *PR2* | GTCTCCTTCGGTGGTAGTG | M37753 | 57 | 104 | Beta 1-3 Endoglucanase | [17, 46] |
|  | ACCCTCCTCCTGCTTTCTC |  |  |  |  |  |
| *PR3* | GCACTTGGTCTGGATTTG | AF202731 | 53 | 115 | Chitinase class I | [17, 46] |
|  | GGCTTGATGGCTTGTTTC |  |  |  |  |  |
| *PR4* | GCTTGCGGGTGACAAATAC | Z11977 | 57 | 96 | Wound-induced protein | [47] |
|  | ACACTCCCACGTCCAAATC |  |  |  |  |  |
| *PR10* | GCCCAGGAACCATCAAGAAG CGCTGTAGCTGTATCCCAAG | AJ289155 | 58 | 108 | Stress-induced ribonuclease-like protein | [47] |
| *PR12* | CATGGACAAGGCACGATTTGG | BU964598 | 62 | 108 | Defensin precursor | [47] |
|  | AACCGATGGCTCTTTGACTCAC |  |  |  |  |  |
| *PAL* | GTGCAAGGGCTGCTTATG CCCAGTCCCTAATTCCTCTC | X52953 | 57 | 107 | Phenylalanine ammonia-lyase | [46,47] |
| *PPO* | GGGTTGGTGCTGCTGATAAG | EF158428 | 62 | 100 | Polyphenol oxidase | [47] |
|  | CGATCCGAGTTCGTGTGATG |  |  |  |  |  |

a NCBI accession number of *Glycine max* gene

b Primer annealing temperature
