# Supplementary material for: Co-Inoculation with Rhizobia and AMF Inhibited Soybean Red Crown Rot: From Field Study to Plant Defense-Related Gene Expression Analysis
Source: PLoS One. 2012 Mar 19;7(3):e33977. doi: 10.1371/journal.pone.0033977 (PMC3307780; doi:10.1371/journal.pone.0033977)
Supplement: Table S2 — Plant N and P content affected by C. parasiticum infection and P level in field. (DOC) [file pone.0033977.s005.doc]

Table S2. Plant N and P content affected by *C. parasiticum* infection

and P level in field.

| P level | Plant P content (mg/plant) | | | | |  | Plant N content (mg/plant) | | | | |
| --- | --- | --- | --- | --- | --- | --- | --- | --- | --- | --- | --- |
|  | Healthy Plant | |  | Infected Plant | |  | Healthy Plant | |  | Infected Plant | |
|  | 2009 | 2010 |  | 2009 | 2010 |  | 2009 | 2010 |  | 2009 | 2010 |
| NP | 82.45±4.68b | 101.56±6.26b |  | 53.51±3.05a | 58.07±3.62a |  | 763.43±61.03b | 952.26±34.85b |  | 425.72±22.81a | 424.96±43.41a |
|  |  |  |  |  |  |  |  |  |  |  |  |
| HP | 111.57±5.74a | 135.20±3.14a |  | 59.56±3.65a | 65.15±4.03a |  | 1040.96±37.72a | 1261.33±69.33a |  | 527.94±39.25a | 562.51±65.92a |

Note: Healthy plant was not infected by *C. parasiticum*; infected plant was infected by *C. parasiticum* with severe necrosis on the subterranean stem and roots, chlorosis of leaves. HP: 80 kg P_2_O_5_ ha^-1^ added as calcium superphosphate, NP: none P fertilizer added. All the data were the mean of four replicates with SE. The same letter after numbers in the same column indicated not significant at 0.05 (*P*＜0.05).
